# Environmental Parasitology: intestinal helminth parasites of the siganid fish *Siganus rivulatus* as bioindicators for trace metal pollution in the Red Sea

**DOI:** 10.1051/parasite/2019014

**Published:** 2019-03-06

**Authors:** Zaki M. Al-Hasawi

**Affiliations:** 1 Biological Sciences Department, Faculty of Science, King Abdulaziz University PO Box 80203 Jeddah 21589 Saudi Arabia

**Keywords:** Siganid fish, Metal pollution, Intestinal helminths, Bioindicators, Red Sea

## Abstract

Studies on host-parasite systems as bioindicators for monitoring trace metal pollution in marine environments are still scarce. Here, 11, 14 and 17 infrapopulations of *Gyliauchen volubilis* (Trematoda: Digenea), *Procamallanus elatensis* (Nematoda) and *Sclerocollum rubrimaris* (Acanthocephala), respectively, were collected from the fish *Siganus rivulatus* (Siganidae) in the Red Sea, from a chronically polluted small bay at Sharm El-Sheikh, South Sinai, Egypt. Water and sediment samples from the bay, tissue samples (intestines, liver, and muscle) from each fish, and samples from each parasite were taken for heavy metal analyses of cadmium and lead. Cadmium concentrations in intestines, liver and muscle of non-infected and infected fishes were much lower than those of lead, and both metal concentrations decreased in the order: liver > intestines > muscle. Cadmium and lead concentrations in the tissues of fishes infected with *G. volubilis* or *P. elatensis* were slightly lower than those in non-infected ones, while in the tissues of fishes infected with *Sc. rubrimaris*, they were much lower. Low concentrations of cadmium and lead in *G. volubilis* and *P. elatensis* are associated with their limited ability or capacity to accumulate trace metals. Therefore, bioconcentration factors corresponding to these species were relatively low, and both are herein considered as weak bioindicators. By contrast, high concentrations of cadmium and lead in *S. rubrimaris* are associated with its high ability to accumulate trace metals. Of the present three host–parasite systems, only the *Siganus rivulatus*–*Sclerocollum rubrimaris* system seems to be promising for biomonitoring of metal pollution in the Red Sea.

## Abstract (fr)

Les études sur les systèmes hôte-parasite utilisés comme bioindicateurs pour surveiller la pollution par les métaux à l’état de traces dans les environnements marins sont encore rares. Ici, 11, 14 et 17 infrapopulations, respectivement de *Gyliauchen volubilis* (Trematoda : Digenea), *Procamallanus elatensis* (Nematoda) et *Sclerocollum rubrimaris* (Acanthocephala), ont été prélevées chez le poisson *Siganus rivulatus* (Siganidae) en Mer Rouge, dans une petite baie chroniquement polluée à Sharm El-Sheikh, Sinaï Sud, Égypte. Des échantillons d’eau et de sédiments de la baie, des échantillons de tissus (intestin, foie, muscles) de chaque poisson et des échantillons de chaque parasite ont été prélevés pour des analyses de métaux lourds (cadmium et plomb). Les concentrations de cadmium dans l’intestin, le foie et le muscle de poissons infectés et non infectés étaient bien inférieures à celles de plomb, et les concentrations de métaux diminuaient dans l’ordre suivant : foie > intestin > muscle. Les concentrations de cadmium et de plomb dans les tissus des poissons infectés par *G. volubilis* ou *P. elatensis* étaient légèrement réduites par rapport à celles des poissons non infectés, alors qu’elles étaient fortement réduites dans les tissus des poissons infectés par *S. rubrimaris*. Les faibles concentrations de cadmium et plomb chez *G. volubilis* et *P. elatensis* sont associées à leur capacité limitée à accumuler des métaux traces. Ainsi, les facteurs de bioconcentration correspondant à ces espèces étaient relativement faibles et les deux sont considérées ici comme des bioindicateurs faibles. Par contre, les fortes concentrations de cadmium et plomb chez *Sc. rubrimaris* sont associées à sa grande capacité à accumuler des métaux à l’état de traces. Parmi les trois systèmes hôte-parasite présents, seul le système *Siganus rivulatus*–*Sclerocollum rubrimaris* semble être prometteur pour le suivi biologique de la pollution par les métaux en Mer Rouge.

## Introduction

Currently, “Environmental Parasitology” is a well-known scientific field concerned with the interactions between parasites and pollutants in the environment [18, 53]. In this interesting field, helminth parasites, especially intestinal ones (digenean trematodes, cestodes, nematodes and acanthocephalans) are used as bioindicators for heavy metal pollution in the environment [18, 53]. These metals include both biologically essential (e.g. Cu, Ni, Zn and Fe) and non-biologically essential or toxic metals (e.g. Cd, Pb and Hg). The first group plays an important role in the metabolic activities of organisms, while the second group is mostly toxic, even in traces under certain conditions [14, 58].

Intestinal helminth parasites of fishes are considered good sentinel organisms for the biomonitoring of heavy metal pollution in aquatic environments [41, 51, 53]. Only a few previous studies have shown that digenetic trematodes of freshwater fishes [49] or marine fishes [21, 30] accumulate some metals in concentrations much lower than those accumulated by acanthocephalans and cestodes [42]. Previous studies have also shown that parasitic nematodes mainly accumulate essential metals rather than toxic ones [6, 39, 53]. However, accumulation of these metals appears to be variable and depends on the nematode species. As a result, some authors consider them to be good sentinels for heavy metal pollution in aquatic environments [33, 35, 45], while other authors consider them to be inappropriate sentinels for toxic metal pollution in these environments, especially if environmental concentrations are low [22, 36, 39, 57]. Controversially, acanthocephalans and cestodes are now well documented as good sentinels for heavy metal pollution in aquatic environments, since they have a high accumulation capacity and are able to accumulate different metals, especially toxic metals at high levels [13, 23, 28, 29, 36, 39, 51–53]. Generally, most research in this field has been carried out to study host-parasite systems in freshwater ecosystems, but those in marine ecosystems are still scarce [40]. To date, only three studies in this field are known from the Red Sea region [8, 22, 23]. In these studies, five monogenean species, four nematode species and one acanthocephalan species, respectively, from Red Sea fishes were considered good sentinels for the biomonitoring of trace metal pollution in this sea.

In the present study, a sample of siganid fish *Siganus rivulatus* was collected from a chronically polluted bay in the Red Sea. In this sample, some fish were parasitized only by an intestinal trematode, some were parasitized only by an intestinal nematode, and some were parasitized only by an intestinal acanthocephalan. Cadmium (Cd) and lead (Pb) concentrations in these three different host–parasite systems were measured to elucidate and to compare the abilities of these different helminths to accumulate heavy metals in their body, and consequently their usefulness as bioindicators for trace-metal pollution in the Red Sea.

## Materials and methods

### Ethics

The sampling reported in this paper complied with the current environmental laws and animal ethics regulations of the Egyptian Environmental Affairs Agency (EEAA).

### Sampling and sample preparation

In June 2018, a sample of 64 specimens of the fish *Siganus rivulatus* (Teleostei, Siganidae), nearly similar in size (13–17 cm in fork length), were caught by a trawling net in the Red Sea, from a small bay (ca. 1 km in diameter) known as El-Mena Bay (boat harbour) (27°51.2′N, 34°17′E), at Sharm El-Sheikh, South Sinai, Egypt. Five water and five sediment samples were taken from different locations along this bay, which is chronically polluted due to massive tourism, and the accumulation of an intensive layer of solid waste and sewage on its bottom, either from land-based activities or other maritime and anthropogenic activities. Additionally, there are about 400 diving boats anchoring in the bay and most of them discharge their wastewater directly into the bay without treatment.

Samples of dorsal muscle, middle intestines and liver were taken from each fish and kept frozen at –20 °C until being processed for metal analysis.

The infrapopulation (all individual worms) of *Gyliauchen volubilis* Nagaty, 1956 (Digenea: Gyliauchenidae), *Procamallanus elatensis* Fusco et Overstreet, 1979 (Nematoda: Camallanidae) or *Sclerocollum rubrimaris* Schmidt et Paperna, 1978 (Acanthocephala: Cavisomidae) found in the intestines of each infected fish was perfectly teased out and carefully counted; 20 worms were taken from each infrapopulation as a representative sample, carefully homogenised into a composite, and kept frozen at –20 °C until being processed for metal analysis.

To reduce the possibility of sample contamination, all the standard precautions were taken during collection and treatment of samples.

### Metal analysis

Water samples were filtered through a 0.45 μm membrane filter and acidified with suprapure HNO_3_ to pH less than 2, then analysed directly for the heavy metals Cd and Pb in an inductively coupled plasma mass spectrometer (ICP-MS-Perkin Elmer ELAN6100). Standards and blanks were processed similarly. Metal concentrations in water samples are expressed as μg L^−1^.

Sediment samples were analysed according to Oregioni and Aston [43]. In brief, samples were dried in an oven at 110 °C for 6 h, and then ground in an agate mortar. One gram of homogenised sample was sieved through a 0.75 mm sieve and digested by a mixture of concentrated acids (HNO_3_/HClO_4_/HF = 3/2/1). The residue was then dissolved in 3% HCl (v/v) and its volume made up to 50 mL in a volumetric flask, and then analysed for the heavy metals in the above*-*mentioned instrument. Blank digestions were processed in the same way. Metal concentrations in sediments are expressed as mg kg^−1^ dry weight.

Fish and parasite tissue samples were analysed according to Zimmermann et al. [60]. After thawing, 150 mg (wet weight) of the homogenised fish tissues or 50 mg of parasites were transferred to a 150 mL perfluoralkoxy (PFA) vessel. A mixture of 2 mL HNO_3_ (65%, suprapure) and 2.5 mL H_2_O_2_ (30%, suprapure) was added and the vessel was heated for 90 min at about 170 °C in a microwave digestion system (CEM GmbH, Kamp-Lintfort, Germany; Model MDS-2000). After digestion, the resulting solution was diluted to 5 mL with high-quality deionised water in a volumetric glass flask, and then analysed for the heavy metals in the above*-*mentioned instruments. Standards and blanks were processed similarly. Metal concentrations in tissues are expressed as mg kg^−1^ wet weight.

Three standard reference materials were used to test the quality of the analytical procedures: 1) CRM–NIST 1640 – Trace Elements in Natural Water-National Institute of Standards and Technology, USA, 2) HISS-1-Marine Sediments – National Research Council, Canada, and 3) Dogfish muscle-DORM2 – National Research Council, Canada. Analytical blanks were prepared to determine the detection limits.

### Data analysis

Linear regression analyses were used to determine possible relationships between metal concentrations in the parasite body and their concentrations in fish intestines, metal concentrations in fish intestines and parasite infrapopulation size, and between metal concentrations in the parasite and its infrapopulation size. The Graph Pad PRISM 7.0 statistical package (GraphPad Software, San Diego, CA 92037, USA) was used for data analyses. The bioconcentration factor (BCF) or the ratio of metal concentration in the parasite and the host tissue (*C*_[parasite]_/*C*_[host tissue]_) was calculated as recommended by Sures et al. [55].

## Results

Of the 64 *Siganus rivulatus* examined, 15 (23.43%) were free from any intestinal helminth parasites, while the other 49 (76.57%) were infected with at least one of three different intestinal helminths: *Gyliauchen volubilis* Nagaty, 1956 (Trematoda: Gyliauchenidae) [41], *Procamallanus elatensis* Fusco et Overstreet, 1979 (Nematoda: Camallanidae) [17], and *Sclerocollum rubrimaris* Schmidt et Paperna, 1978 (Acanthocephala: Cavisomidae) [48]. All patterns of single and concurrent infections (double or triple) with these parasites were found in the infected fishes: 11 (17.19%) with *G. volubilis* only, 14 (21.88%) with *P. elatensis* only, 17 (26.56%) with *S. rubrimaris* only, 3 (4.69%) with *G. volubilis* and *P. elatensis*, 1 (1.56%) with *G. volubilis* and *S. rubrimaris*, 2 (3.13%) with *P. elatensis* and *S. rubrimaris*, and 1 (1.56%) with *G. volubilis*, *S. rubrimaris* and *P. elatensis*. Fishes with single infection contained large numbers of worms (32–210 worm/fish), while those with concurrent infections contained few numbers of worms (4–13 worm/fish) and were excluded from the study to avoid confusion.

Accordingly, 11, 14 and 17 infrapopulations of *G. volubilis*, *P. elatensis* and *S. rubrimaris* ranging from 63 to 159, 55 to 179 and from 32 to 210 individuals, respectively, were collected from the infected fishes for metal analyses.

Cadmium and lead concentrations recovered from standard reference materials, the accuracy of the analytical procedures, and the detection limits of each element are shown in Table 1.

**Table 1:** Cadmium and lead concentrations in certified reference materials, and accuracy and detection limits determined by ICP-MS analyses.

Metal	Standard reference material									Detection limit mg kg^−1^
	CRM–NIST 1640 – Trace elements in natural water			HISS-1 – Marine sediments			Dogfish muscle-DORM2			
	Certified value (mg L^−1^)	Recovered value (mg L^−1^)	Accuracy (%)	Certified value (mg kg^−1^ dry wt)	Recovered value (mg kg^−1^ dry wt)	Accuracy (%)	Certified value (mg kg^−1^ wet wt)	Recovered value (mg kg^−1^ wet wt)	Accuracy (%)	
Cd	3.961 ± 0.072	3.848 ± 0.064	97.14	0.024 ± 0.009	0.022 ± 0.003	91.66	0.043 ± 0.008	0.042 ± 0.003	97.67	0.003
Pb	12.005 ± 0.040	11.526 ± 0.105	99.00	3.140 ± 0.04	3.008 ± 0.057	95.79	0.065 ± 0.007	0.063 ± 0.006	96.92	0.006

### Cd and Pb concentrations in water and sediment samples from the bay

Mean Cd and Pb concentrations in the sediments were much greater than those in water (Table 2), and permanent Pb concentrations were greater than those of Cd.

**Table 2:** Range and mean Cd and Pb concentrations in the water and sediments at five sites in El-Mena Bay (Red Sea).

	Water (μg L^−1^)		Sediment (mg kg^−1^ dry wt)	
	Cd	Pb	Cd	Pb
Range	0.097–0.118	3.142–4.261	0.295–0.402	9.112–13.315
Mean ± SD	0.107 ± 0.008	3.769 ± 0.492	0.341 ± 0.039	10.932 ± 1.646

### Cd and Pb concentrations in selected tissues of non-infected and infected fishes

Mean Cd and Pb concentrations in the intestines, liver and muscle of non-infected and infected individuals of the fish *S. rivulatus* (with *G. volubilis*, *P. elatensis* or with *S. rubrimaris*) are shown in Table 3. In all cases, mean Cd concentrations in these tissues were much lower than those of Pb, and concentrations of both metal decreased in the order: liver > intestines > muscle.

**Table 3:** Range and mean Cd and Pb concentrations in the selected tissues of non-infected and infected individuals of *Siganus rivulatus* (with *Gyliauchen volubilis*, *Procamallanus elatensis* or *Sclerocollum rubrimaris*).

Fish	Parasite infrapopulation size	Cd (mg kg^−1^ wet wt)				Pb (mg kg^−1^ wet wt)			
		Range (mean ± SD)				Range (mean ± SD)			
		Parasite	Fish (*S. rivulatus*) tissues			Parasite	Fish (*S. rivulatus*) tissues		
			Intestines	Liver	Muscle		Intestines	Liver	Muscle
Non-infected (*n* = 15)	–	–	0.068–0.138	0.178–0.349	0.043–0.082	–	0.257–0.732	1.239–2.641	0.102–0.178
			(0.105 ± 0.021)	(0.259 ± 0.050)	(0.061 ± 0.012)	–	(0.494 ± 0.132)	(1.924 ± 0.167)	(0.132 ± 0.024)
Infected with *G. volubilis* (*n* = 11)	63–159	*G. volubilis*				*G. volubilis*			
		0.088–0.171	0.061–0.124	0.192–0.282	0.041–0.074	0.460–0.872	0.242–0.706	1.202–2.485	0.094–0.159
		(0.132 ± 0.023)	(0.094 ± 0.021)	(0.235 ± 0.031)	(0.056 ± 0.011)	(0.656 ± 0.130)	(0.463 ± 0.158)	(1.715 ± 0.386)	(0.124 ± 0.018)
Infected with *P. elatensis* (*n* = 14)	55–179	*P. elatensis*				*P. elatensis*			
		0.106–0.211	0.056–0.117	0.182–0.264	0.038–0.075	0.471–0.883	0.332–0.684	1.123–2.345	0.086–0.145
		(0.138 ± 0.026)	(0.087 ± 0.021)	(0.228 ± 0.025)	(0.053 ± 0.011)	(0.692 ± 0.114)	(0.458 ± 0.014)	(1.694 ± 0.346)	(0.114 ± 0.019)
Infected with *S. rubrimaris* (*n* = 17)	32–210	*S. rubrimaris*				*S. rubrimaris*			
		10.505–18.193	0.027–0.075	0.085–0.148	0.012–0.041	15.382–26.382	0.132–0.438	0.739–1.107	0.017–0.053
		(13.713 ± 2.561)	(0.047 ± 0.016)	(0.115 ± 0.020)	(0.029 ± 0.009)	(20.610 ± 3.660)	(0.270 ± 0.093)	(0.896 ± 0.114)	(0.030 ± 0.010)

As shown in Table 3, mean Cd and Pb concentrations in the intestines, liver and muscle of fishes infected with *G. volubilis* or with *P. elatensis* were slightly lower than those in non-infected fishes, but in fishes infected with *S. rubrimaris* these concentrations were much lower than those in non-infected fishes. These results clearly indicate that metal accumulation in fish tissues was slightly reduced due to infection with the trematode *G. volubilis* or with the nematode *P. elatensis*, but dramatically reduced due to infection with the acanthocephalan *S. rubrimaris*.

### Cd and Pb concentrations in helminth parasites

**Figure 1:**
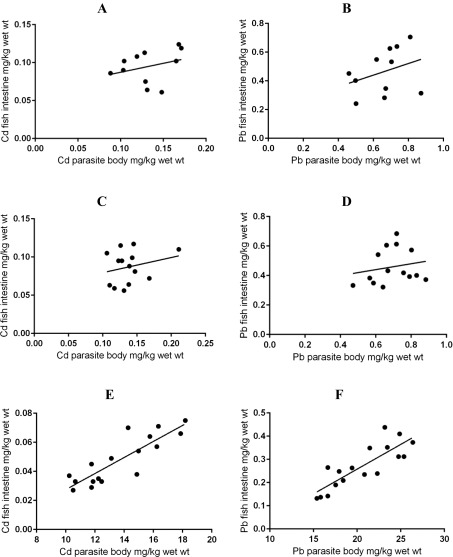
The relationships between metal concentrations in fish intestines and their concentrations in intestinal helminth parasites: (A) Cd concentrations in fish intestines vs. concentrations in *G. volubilis*; (B) Pb concentrations in fish intestines vs. concentrations in *G. volubilis*; (C) Cd concentrations in fish intestines vs. concentrations in *P. elatensis*; (D) Pb concentrations in fish intestines vs. concentrations in *P. elatensis*; (E) Cd concentrations in fish intestines vs. concentrations in *S. rubrimaris*; and (F) Pb concentrations in fish intestines vs. concentrations in *S. rubrimaris.*

**Figure 2:**
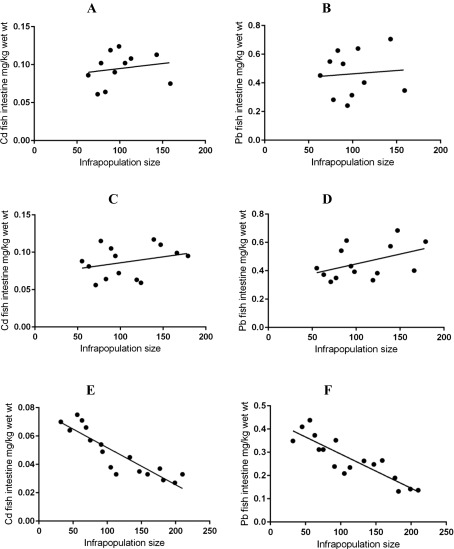
The relationships between metal concentrations in the intestines of infected fish and parasite infrapopulation size in its intestines. (A) Cd concentrations in fish intestines vs. *G. volubilis* infrapopulation size; (B) Pb concentrations in fish intestines vs. *G. volubilis* infrapopulation size; (C) Cd concentrations in fish intestines vs. *P. elatensis* infrapopulation size; (D) Pb concentrations in fish intestines vs. *P. elatensis* infrapopulation size; (E) Cd concentrations in fish intestines vs. *S. rubrimaris* infrapopulation size; and (F) Pb concentrations in fish intestines vs. *S. rubrimaris* infrapopulation size.

**Figure 3:**
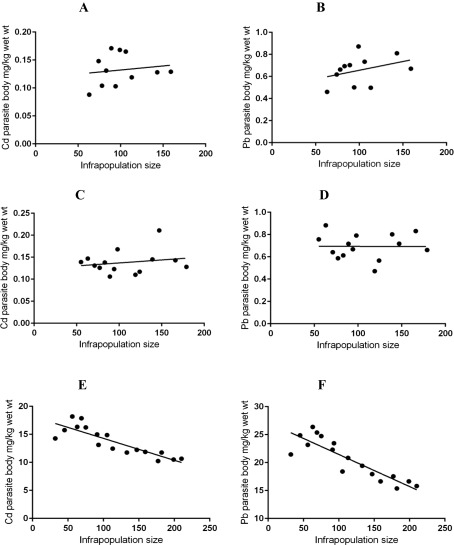
The relationships between metal concentrations in parasites and infrapopulation size in fish intestines: (A) Cd concentrations in *G. volubilis* vs. infrapopulation size; and (B) Pb concentrations in *G. volubilis* vs. infrapopulation size; (C) Cd concentrations in *P. elatensis* vs. infrapopulation size; (D) Pb concentrations in *P. elatensis* vs. infrapopulation size; (E) Cd concentrations in *S. rubrimaris* vs. infrapopulation size; and (F) Pb concentrations in *S. rubrimaris* vs. infrapopulation size.

Cadmium and Pb concentrations were calculated for 11, 14 and 17 infrapopulations of *G. volubilis*, *P. elatensis* and *S. rubrimaris*, respectively (Table 3). As shown in this table, mean Cd and Pb concentrations in the body of *G. volubilis* or in the body of *P. elatensis* were slightly higher than those in the intestines of their fish hosts. However, no significant relationships were found between Cd or Pb concentrations in *G. volubilis* and their concentrations in fish intestines (*R* ^2^ = 0.09458, slope = 0.2352, *p* = 0.3576; *R* ^2^ = 0.1105, slope = 0.4044, *p* = 0.3179, respectively) (Fig. 1A and B), or between Cd or Pb concentrations in *P. elatensis* and their concentrations in fish intestines (*R* ^2^ = 0.06052, slope = 0.1979, *p* = 0.3965; *R* ^2^ = 0.0364, slope = −0.200, *p* = 0.5136, respectively) (Fig. 1C and D). Also, no significant relationships between Cd and Pb concentrations in fish intestines and *G. volubilis* infrapopulation size were found (*R* ^2^ = 0.03252, slope = 0.00013, *p* = 0.5957; *R* ^2^ = 0.00736, slope = 0.00046, *p* = 0.8014, respectively) (Fig. 2A and B), or between Cd or Pb concentrations in *G. volubilis* and its infrapopulation size (*R* ^2^ = 0.02295, slope = 0.00014, *p* = 0.6591; *R* ^2^ = 0.1265, slope = 0.00158, *p* = 0.283, respectively) (Fig. 3A and B). Similarly, no significant relationships between Cd and Pb concentrations in fish intestines and *P. elatensis* infrapopulation size were demonstrated (*R* ^2^ = 0.08486, slope = 0.00015, *p* = 0.3123; *R* ^2^ = 0.2045, slope = −0.00139, *p* = 0.1045, respectively) (Fig. 2C and D), or between Cd or Pb concentrations in *P. elatensis* and its infrapopulation size (*R* ^2^ = 0.04015, slope = 0.00014, *p* = 0.2922; *R* ^2^ = 0.00124, slope = −0.00185, *p* = 0.9905, respectively) (Fig. 3C and D). These non-significant relationships mean that there is no evidence for interspecific competition (between the fish host and *G. volubilis* or *P. elatensis*) or intraspecific competition (among parasite individuals) for accumulating these metals. Moreover, mean Cd and Pb concentrations in *G. volubilis* and *P. elatensis* were distinctly lower than those in the fish liver, and a few-fold higher than those in fish muscle (Table 4). A combination of the results clearly indicates that both *G. volubilis* and *P. elatensis* have a limited ability to accumulate heavy metals in their bodies.

**Table 4:** Range and mean bioconcentration factors-BCF [= (*C*_[parasite]_/*C*_[host tissue]_)] for Cd and Pb in 11, 14 and 17 infrapopulations of *Gyliauchen volubilis*, *Procamallanus elatensis* and *Sclerocollum rubrimaris*, respectively, calculated with respect to the selected host tissues.

Fish	Parasite infrapopulation size		BCF = (C_[parasite]_/C_[host tissue]_)			BCF = (C_[parasite]_/C_[host tissue]_)		
			Cd			Pb		
			C_parasite_/*C*_fish intestines_	C_parasite_/*C*_fish liver_	C_parasite_/*C*_fish muscle_	C_parasite_/*C*_fish intestines_	C_parasite_/*C*_fish liver_	C_parasite_/*C*_fish muscle_
Infected with *G. volubilis* (*n* = 11)	63–159	Range	1.0–2.4	0.3–0.8	1.6–3.3	1.0–2.7	0.2–0.5	3.1–7.3
		Mean ± SD	1.4 ± 0.4	0.5 ± 0.1	2.3 ± 0.5	1.5 ± 0.1	0.4 ± 0.1	5.4 ± 1.4
Infected with *P. elatensis* (*n* = 14)	55–179	Range	1.0–2.3	0.4–0.8	1.7–3.9	1.0–2.3	0.2–0.7	3.7–8.5
		Mean ± SD	1.6 ± 0.4	0.6 ± 0.1	2.6 ± 0.7	1.5 ± 0.4	0.4 ± 0.1	6.2 ± 1.5
Infected with *S. rubrimaris* (*n* = 17)	32–210	Range	203.8–405.6	104.1–147.9	367.5–980.2	52.9–117.1	20.1–28.6	469.3–1078.7
		Mean ± SD	303.3 ± 63.6	119.3 ± 12.8	507.0 ± 163.7	81.9 ± 20.1	22.9 ± 2.6	731.8 ± 160.7

Unlike those in *G. volubilis* and *P. elatensis*, Cd and Pb concentrations in *S. rubrimaris* were significantly high and much higher, respectively than those in the intestines of its fish host (Table 4). However, there were significant positive relationships between Cd or Pb concentrations in *S. rubrimaris* and their concentrations in fish intestines (*R* ^2^ = 0.7414, slope = 0.00552, *p* < 0.0001; *R* ^2^ = 0.6915, slope = 0.02132, *p* < 0.0001, respectively) (Fig. 1E and F), i.e. as the Cd and Pb concentrations in fish intestines increased, their concentrations in the parasite body increased. There were strong negative relationships between Cd or Pb concentrations in fish intestines and *S. rubrimaris* infrapopulation size (*R* ^2^ = 0.8026, slope = −0.00026, *p* < 0.0001; *R* ^2^ = 0.7742, slope = −0.001469, *p* < 0.0001, respectively) (Fig. 2E and F), i.e. as the infrapopulation size of *S. rubrimaris* in the intestines of *S. rivulatus* increases, the concentrations of both Cd and Pb in fish intestines significantly decrease. Also, there were strong negative relationships between Cd or Pb concentrations in *S. rubrimaris* and its infrapopulation size (*R* ^2^ = 0.7383, slope = −0.03915, *p* < 0.0001; *R* ^2^ = 0.7749, slope = −0.05733, *p* < 0.0001, respectively) (Fig. 3E and F), i.e. as the infrapopulation size of *S. rubrimaris* increased, the concentration of both Cd and Pb in its individuals significantly decreased. Therefore, metal concentrations in this acanthocephalan seem to be mainly associated with those in fish intestines and with its infrapopulation size. Generally, high concentrations of Cd and Pb in the body of *S. rubrimaris* clearly indicate that this acanthocephalan has a high ability or capacity to accumulate heavy metals in its body.

Mean Cd and Pb concentrations in the body of *G. volubilis* or *P. elatensis* were slightly higher than those in fish intestines, distinctly lower than those in fish liver, and a few-fold higher than those in fish muscle. As a result, bioconcentration factors corresponding to these parasites (Table 4) were relatively low and seemed to be insufficiently large or not sufficiently significant to consider them good bioindicators. In contrast, Cd and Pb concentrations in the body of *S. rubrimaris* were significantly higher than those in the intestines, liver and muscle of its fish host (Table 4). Therefore, the bioconcentration factors corresponding to *S. rubrimaris* seemed to be highly significant, since Cd concentrations in this acanthocephalan reached 405-, 147- and 980-fold higher than in fish intestines, liver and muscle, respectively, while those of Pb reached to 117-, 28- and 1087-fold higher than in these tissues, respectively.

## Discussion

The siganid fish *Siganus rivulatus* and its intestinal helminth parasites, *Gyliauchen volubilis*, *Procamallanus elatensis* and *Sclerocollum rubrimaris* are common in the Red Sea [1–3, 15, 17, 41, 48]. As expected, these parasites have different abilities or capacities to accumulate trace metals in their bodies (see below).

In El-Mena Bay, mean Cd and Pb concentrations in the sediments were much greater than those in water. This is mostly because metals in aquatic environments are usually bound to suspended particles or adsorbed to particulate organic matter, which finally settle and accumulate in bottom sediments [10, 12, 20, 34, 56]. Therefore, metal concentrations are higher in sediment and usually exceed those of the overlying water by 3–5 orders of magnitude [20].

In the current study, Cd concentrations in the selected tissues of both non-infected and infected fishes were much lower than those of Pb, and concentrations of both metals decreased in the order: liver > intestines > muscle. Metals mainly accumulated in metabolically active organs (e.g. liver, kidney, gills and intestines) [16, 31, 32], and accumulated less in organs with relatively low metabolic activity (e.g. muscles) [4, 11, 25, 46]. High concentrations of Cd and Pb in the hepatic tissue can possibly be attributed to its essential role in the accumulation, storage and detoxification of metals [9, 26, 38, 59].

Metals absorbed by the fish through its gills or its intestinal wall are carried by blood to the liver, where most of them are taken from blood to form organometallic complexes transferred with bile (bile complexes) into the small intestine, where they can be re-absorbed through the intestinal wall to enter the hepatic-intestinal cycle, or expelled with the fish faeces [19, 27, 54]. Intestinal helminths interfere with this cycle and ingest/absorb bile complexes according to their ability or capacity to do this [54]. In our study, both *G. volubilis* and *P. elatensis* contained Cd and Pb concentrations slightly higher than those in the intestines of their host fishes, and showed non-significant relationships with them. Thus, these parasites ingest/absorb small amounts of bile complexes from the fish intestines. These amounts have very little effect on those that are normally absorbed across the intestinal wall of the fish, since Cd and Pb concentrations in the tissues of fishes infected with these parasites were slightly reduced compared to non-infected conspecifics. These results agree implicitly with some of the previous studies showing that intestinal trematodes [21, 30, 42, 49] and nematodes [36, 39, 53, 57] of fishes have limited abilities or capacities to accumulate trace metals in their bodies; some authors considered nematodes good sentinels for metal pollution in aquatic environments [7, 33, 35, 44].

Unlike *G. volubilis* and *P. elatensis*, *S. rubrimaris* contained Cd and Pb concentrations much higher than those in the fish intestines, and showed significant positive relationships with them. Acanthocephalans have a strong ability and large capacity to absorb bile complexes via their tegument with higher efficiency than the intestinal wall of the fish host [50, 54]. Consequently, the amount of bile complexes that are normally re-absorbed by the intestinal wall is considerably reduced, and thereby, Cd and Pb concentrations in the intestines, liver and muscle of acanthocephalan-infected fishes were dramatically reduced compared to non-infected conspecifics. Acanthocephalans are now well documented as good sentinels for trace metal pollution in aquatic environments [13, 23, 28, 29, 36, 39, 51–53].

In the present study, no significant relationships were found between Cd and Pb concentrations in fish intestines and *G. volubilis* or *P. elatensis* infrapopulation size, or between Cd and Pb concentrations in *G. volubilis* or *P. elatensis* and its infrapopulation size. This showed that there is no evidence for interspecific competition (between the fish host and *G. volubilis* or *P. elatensis*) or for intraspecific competition (among parasite individuals) for accumulating these metals. There were clear negative relationships between Cd or Pb concentrations in fish intestines and *S. rubrimaris* infrapopulation size, i.e. as the infrapopulation size of *S. rubrimaris* in the intestines of *S. rivulatus* increases, the concentrations of both Cd and Pb in the intestines significantly decrease. This decrease with the increase in infrapopulation size strongly suggests interspecific competition (between the fish host and its acanthocephalan parasite) for accumulating these metals. There were also clear negative relationships between Cd or Pb concentrations in *S. rubrimaris* and its infrapopulation size, i.e. as the infrapopulation size of *S. rubrimaris* increased, the concentration of both Cd and Pb in its individuals significantly decreased. This decrease with the increase in infrapopulation size strongly suggests intraspecific competition (between parasite individuals) for absorbing available metals from fish intestines [57]. Such competition for host limited resources is common in acanthocephalan infrapopulations [24, 45, 47].

Because both *G. volubilis* and *P. elatensis* accumulate few amounts of metals in their bodies, bioconcentration factors corresponding to them were significantly low, and both are considered herein as weak bioindicators for trace metal pollution in marine environments. In contrast, *S. rubrimaris* accumulates significantly higher amounts of metal in its body. Therefore, bioconcentration factors corresponding to this acanthocephalan seemed to be highly significant, since Cd concentrations in its body reached 405-, 147- and 980-fold higher than in fish intestines, liver and muscle, respectively, while those of Pb reached to 117-, 28- and 1087-fold higher than in these tissues, respectively. These values appear to be highly significant, since in marine environments only minute amounts of trace metals are found as free ions (hydrated) or biologically available for uptake by organisms [37].

Of the present three host–parasite systems, *S. rivulatus*–*G. volubilis*, *S. rivulatus*–*P. elatensis* and *S. rivulatus*–*S. rubrimaris*, only the later seems promising for biomonitoring of metal pollution in the Red Sea. *Sclerocollum* is a small acanthocephalan genus and currently includes three species [5]: *S. robustum* (Edmonds 1964) Schmidt et Paperna 1978, *S. rubrimaris* Schmidt et Paperna 1978, and *S. saudii* Al-Jahdali 2010. The second and the third are endemic to the Red Sea. Recently and as in the present study, *S. saudii* was considered a good sentinel for biomonitoring trace metal pollution in the Red Sea [23].

Finally, our results are completely consistent with the previous studies [13, 39, 42, 52, 53] showing that gutless helminth parasites which take up their nutrients via their tegument from fish intestines, such as acanthocephalans and cestodes, are more appropriate sentinels for trace metal pollution than other helminths which have a gastro-intestinal tract, i.e. trematodes and nematodes.

## Competing interests

The author declares that he has no conflict of interest.

## Funding

We are very grateful to King Abdulaziz University, Saudi Arabia, for continued encouragement and support.
